# Impact of COVID-19 pandemic on athletes with disabilities preparing for the Paralympic Games in Tokyo

**DOI:** 10.1186/s13104-021-05646-0

**Published:** 2021-06-14

**Authors:** Piotr Urbański, Łukasz Szeliga, Tomasz Tasiemski

**Affiliations:** 1 Department of Adapted Physical Activity, Poznan University of Physical Education, Poznań, Poland; 2 Polish Paralympic Committee and Polish Sports Association for the Disabled ’Start’, Warsaw, Poland

**Keywords:** Athletic performance, COVID-19, Lockdown, Paralympic Games, Physical disability, Paralympic athletes

## Abstract

**Objective:**

The main aim of the study was to assess the impact of COVID-19 pandemic on athletes preparing for the Tokyo 2021 Paralympic Games during 1 month of lockdown in Poland. The study involved 166 athletes (106 male, 66 female), members of either the Polish Paralympic Committee or the Polish Sports Association for the Disabled’Start’, two organizations responsible for managing and regulating sports played by persons with disabilities in Poland.

**Results:**

Athletes with disabilities have been strongly affected by the pandemic and the resultant lockdown. The majority of respondents reported that they trained at home (88.6%), whereas 60.2% of athletes trained outdoors, and 12% suspended their training regimens altogether. Only 5.4% of athletes had some access to sport facilities. The athletes reduced their weekly training time by almost half (9.4 h/week vs. 5.3 h/week), a statistically significant difference (t = 16.261, p < 0.001).

**Supplementary Information:**

The online version contains supplementary material available at 10.1186/s13104-021-05646-0.

## Introduction

**Fig. 1:**
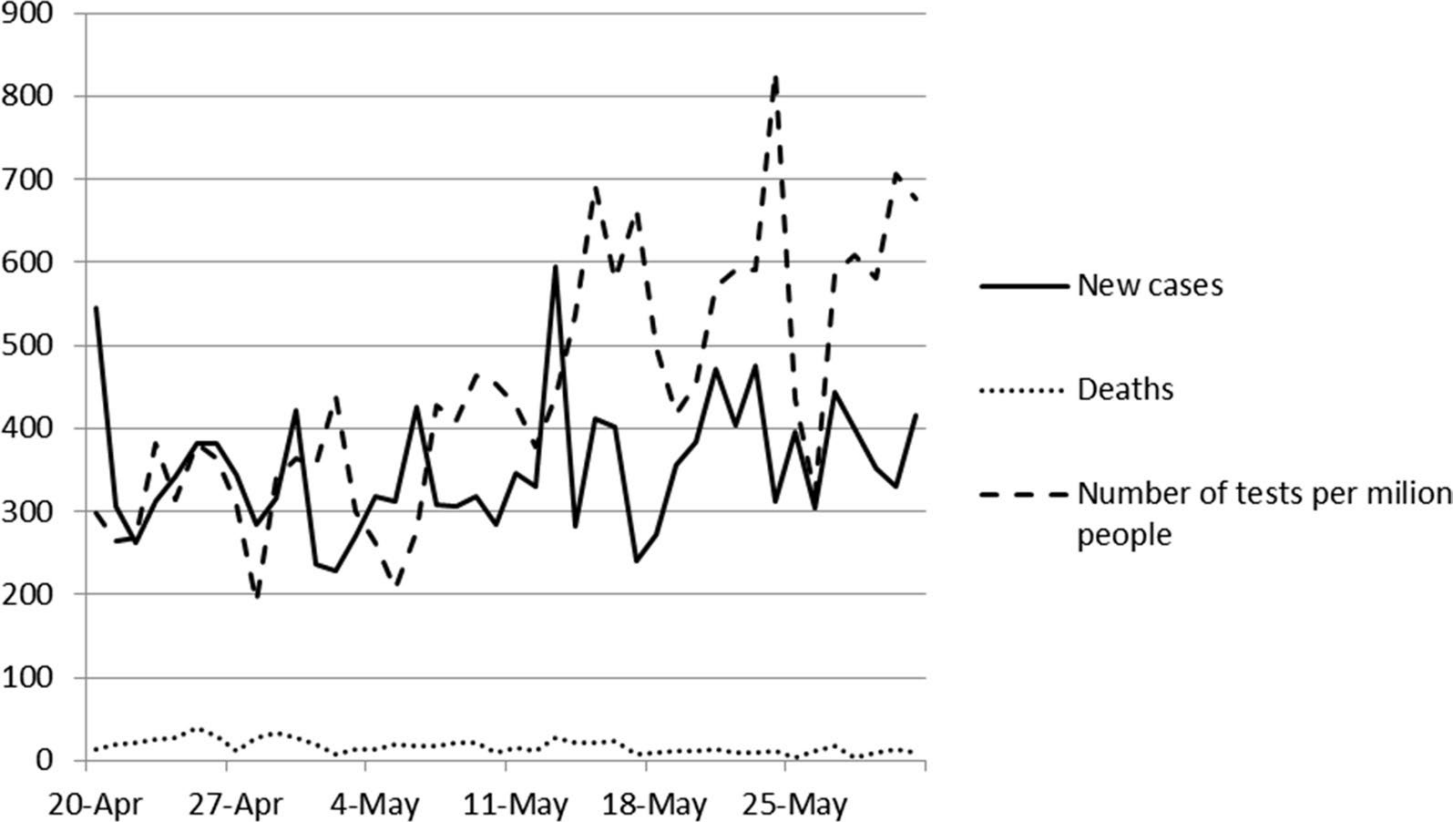
Development of COVID-19 in Poland between April 20 and May 30, 2020

From the end of 2019 onwards, the COVID-19 pandemic caused by the SARS CoV-2 (Severe Acute Respiratory Syndrome Coronavirus) has affected all spheres of human activity, including mostly healthcare system but also economy, tourism, education and sport. Geographically, the pandemic has impacted all regions of the world. By mid-March 2020, Europe had become the epicenter of the epidemic, reporting over 40% of globally confirmed cases; as of 28 April 2020, 63% of global demises attributable to the virus were reported from this WHO Region [1]. In Poland, the government data for the period between March 20 and April 30, 2020 record 14,826 diagnosed COVID-19 cases and 714 resultant deaths (Fig. 1). Many European countries introduced diverse countermeasures against the spread of infection, including the so-called lockdowns of variable severity. In early April 2020, the extent of lockdown measures varied from country to country, with some banning any kind of interhousehold mingling and outdoor activities, some allowing certain types of activities (e.g. Poland, Germany and the UK) and some merely recommending precautionary social distancing measures (Sweden) [2].

The introduction of countermeasures against the spread of COVID-19 and fear linked with the pandemic resulted in cancellation or suspension of almost all sport activities and events around the world, including the 2020 Olympic and Paralympic Games in Tokyo. In effect, the pandemic negatively impacted sport from grassroots physical activity to elite sport events [3]. The limits imposed on physical activity are predicted to negatively impact the health and future performance of athletes living under lockdowns [4]. The enforced isolation is expected to lead to cessation of organized training/competitive activities and prolonged periods of inactivity, broken up by rare training sessions in suboptimal conditions (no or limited access to training facilities and equipment) [2, 3, 5]. Team relationships will likely deteriorate due to limited socializing opportunities between athletes, coaches and team support networks. Finally, the limits imposed on travel will further impact athletes with disabilities who were already suffering from mobility issues attributable to inadequate public transport infrastructure designed to accommodate people with disabilities [6, 7].

Scholarly reports published so far on the pandemic’s impact have assessed the influence of COVID-19 on the performance of athletes, underlining the need for regular physical activity during the period of social isolation [5, 8, 9]. Some of the studies report that the influence of pandemic is differentiated, 40.5% of inactive individuals became even less active, and only 22.4% of active individuals became less active. Comparatively, 33% of inactive individuals became more active while 40.3% of active individuals became even more active [10]. Other studies indicate that the decreased level of physical activity of children and adults living under lockdowns may expose them to an increased risk of obesity, associated with various immediate and long-term comorbidities, such as sleep apnea, hypertension, type 2 diabetes, heart disease, stroke and reduced immunity [5, 11]. The psychophysiological conditions of life under lockdown and in quarantine may negatively affect the activity of the sympathetic nervous system: the uncertainty and isolation may result in abnormally prolonged release of cortisol and catecholamine, potentially leading to different pathologies and psychopathologies, such as anxiety, depression, loneliness or sleep disorders [6, 12].

Considering social repercussions of the COVID-19 pandemic, persons with disabilities constitute a distinct group in regard to their health conditions [13] and health risks [14]. The studies demonstrate that people with specific comorbidities associated with disabilities (e.g. impaired immune function, either by disease or medication, renal/hepatic dysfunction, cardiovascular diseases, lung conditions) may exhibit more serious symptoms when suffering from the COVID-19 [15, 16] and therefore require additional safety measures, such as stringent social isolation. Nevertheless, it is well known that physical activity, sport and socializing play a key role in physical rehabilitation of people with disabilities [17]. Participation in physical activity and sport measurably increases their self-sufficiency and psychophysical resilience. Benefits of physical activity are often not deemed significant enough to justify relaxing social distancing rules in the context of the perceivably greater threat of COVID-19. The above holds especially true for athletes with disabilities who train for competitions under the lockdown.

Although one study [18] demonstrated that time spent engaging in sedentary screen time activities increased during the COVID-19 pandemic, it also highlighted the general resilience of athletes, with no discernible differences in training, fitness, or dietary intake over a short (3 months) period. Nevertheless, this research covers only one specific discipline and the topic demands further investigation. During March and June 2020 in Poland, all sports facilities and gyms were closed due to governmental mitigation measures (social isolation and social distancing). In result, athletes could only train outdoors, most often individually, often without any direct input from their coach. Since persons with disabilities often need support of their caregivers, athletes with disabilities face unique difficulties when forced to train individually.

In light of the situation described above, the main aim of the study was to assess the situation of athletes with disabilities preparing for the Tokyo 2021 Paralympic Games during the lockdown period (from March 20 to April 30, 2020) in Poland. We were particularly interested in necessary modifications in training caused by the governmental countermeasures against the COVID-19 pandemic.

## Main text

### Methods

#### Survey design

The questionnaire was prepared in cooperation with the European Paralympic Committee and Polish Paralympic Committee, including the input of sports section coaches, a sports psychologist and a physiotherapist (Additional file 1). The questionnaire has been designed so that the respondents can complete it quickly and without undue effort. The survey included questions about the effect of the COVID-19 on the average training time during the pandemic (in relation to the original training schedule), satisfaction with training, accessibility of sport facilities and other aspects of sports performance.

#### Procedures

Study participants were registered members of the Polish Paralympic Committee or the Polish Sports Association for the Disabled’Start’, two organizations responsible for managing and regulating sports played by persons with disabilities in Poland. All participants were training in preparation for the Paralympic Games in Tokyo. Participants were recruited in May 2020 and invited to complete a cross-sectional survey. During the first week of June, 200 athletes were electronically sent a link to an online survey hosted by Google Forms, with completion reminders sent weekly during the next two weeks. A total of 188 surveys were completed (response rate 94%), with 22 surveys excluded due to missing data: in result, the study considered data gathered from 166 surveys.

#### Participants

Male respondents constituted the majority of the study participants (63.9%). The main type of disability reported by participating Paralympic athletes was spinal cord injury i.e., paraplegia and tetraplegia (25.9%), followed by limb amputations (22.2%). The mean age of study participants was 33 years (SD = 11.7), and the mean time since injury or diagnose of disease was 20 years (SD = 12.5). Study participants represented 15 Paralympic sport disciplines. The most commonly represented sport was athletics (23.5%), whereas the average training experience in years amounted to 10 (SD = 7.0). The respondents’ characteristics are presented in Table 1.

**Table 1:** Socio-demographic and injury characteristic of 166 athletes with disabilities

Socio-demographic and injury characteristic	Athletes with disabilities (n = 166)	
	n	(%)
Gender		
Male	106	(63.9)
Female	60	(36.1)
Marital status		
Single	105	(63.3)
In relationship	61	(24.1)
Place of living		
Urban area	126	(76.5)
Rural area	39	(23.5)
Education		
Higher	160	(96.4)
Elementary/vocational	6	(3.6)
Professional status		
Employed	146	(88.0)
Unemployed	20	(12.0)
Sport discipline		
Archery	7	(4.2)
Athletics	39	(23.5)
Boccia	4	(2.4)
Canoe	2	(1.2)
Cycling	7	(4.2)
Goalball	9	(5.4)
Powerlifting	15	(9.0)
Rowing	6	(3.6)
Shooting	5	(3.0)
Swimming	25	(15.1)
Sitting volleyball	11	(6.6)
Table tennis	10	(6.0)
Wheelchair fencing	5	(3.0)
Wheelchair rugby	8	(4.8)
Wheelchair basketball	13	(7.8)
Type of disability		
Paraplegia	21	(12.6)
Tetraplegia	22	(13.3)
Muscular dystrophy	3	(1.8)
Heine-Medina (poliomyelitis)	2	(1.2)
Spina bifida	10	(6.0)
Cerebral palsy	14	(8.4)
Limb amputations	37	(22.2)
Multiple sclerosis	2	(1.2)
Other	55	(33.1)

#### Statistical analysis

Descriptive data was presented as *n*, *percent*, *mean* and *standard deviation* (*SD*). To assess the difference between planned and executed training time, the independent samples t Test was performed. All statistical analyses were performed with the IBM Statistical Package for Social Sciences software (IBM SPSS Statistics version 21, Chicago, IL, USA). The level of significance was set at *p* ≤ 0.05.

### Results and discussion

The COVID-19 pandemic has affected athletes with disabilities and sports institutions all over the word. A new challenge in the contemporary world of sport, the pandemic demanded and continues to demand extraordinary flexibility and resilience from sport managers, who have to adapt to rapidly shifting conditions. This study’s aim was to assess the situation and training opportunities for elite athletes with disabilities preparing to participate in the Tokyo Paralympic Games 2021 and to indicate relevant factors that could hamper their performance during this elite sport event.

The results of this study showed that the vast majority of athletes were affected by the pandemic and/or governmental countermeasures such as lockdowns. Even though during the study period only 9% of athletes were quarantined and 1.8% of athletes and 0.6% of coaches contracted COVID-19, the social distancing measures almost universally affected athletes’ training regimens (e.g. closed venues/gyms or limitations for total number of people at sport venues). The majority of respondents reported that they trained at home (88.6%), 60.2% of athletes trained outdoors, and 12% suspended their training regimens altogether. Only 5.4% of athletes had some form of access to sport facilities. On average, the athletes reduced their weekly training time by almost half (9.4 h/week vs. 5.3 h/week), a statistically significant difference (t = 16.261, p < 0.001). In addition, all sports and training camps were canceled during the study period, with 60.8% of study participants reporting difficulties due to insufficient contact with assistants/caregivers or due to lack of access to assistive training devices. Over 74% of athletes were not satisfied with their abilities to train during the pandemic.

The sport of people with disabilities undergoes rapid professionalization [19]. For a long time, an erroneous tendency existed to conduct research on athletes with disabilities with the same mindset as the one applied to studies on non-disabled athletes. Today, it is known that sport plays a special role for persons with disabilities, increasing their socialization, self-esteem, quality of life and independence [17, 20]. Therefore, it is even more important to continue monitoring the pandemic’s influence on athletes with disabilities preparing for elite sport events.

The present study was conducted in cooperation with the European Paralympic Committee and the Polish Paralympic Committee. Its practical goal was to promote effective cooperation in order to produce and exchange information about feasible solutions which could be beneficial in similar future scenarios. The survey was conducted in the initial phase of the epidemic in Poland, when the number of reported infections did not exceed five hundred cases a day. As of January 6, 2021, the official data given by the Polish government indicate that over 1,300,000 people in Poland have become infected with and more than 30,000 died due to COVID-19, with the highest daily increase in the number of infections amounting to about 30,000 [1].

### Conclusions

The strength of this study resides in very high response rate and capturing the most restricted period of pandemic in Poland. Almost all Polish Paralympic Athletes preparing to Paralympic Games Tokyo 2021 took part in this study during period were all sports facilities were closed, and even ability to train outdoor was very limited. The present study showed that the Polish Paralympic athletes with disabilities have been strongly affected by the pandemic and measures undertaken to slow its spread, especially by the lockdown. Since they were often prevented from regular training, their preparation process for the Paralympic Games in Tokyo was disturbed during the observed 1 month of pandemic lockdown. Further longitudinal studies with cooperation of the National Paralympic Committees and other sports institutions are definitely warranted to counteract the effects of the pandemic on athletes with disabilities.

## Limitations

There are some limitations to the present study. First, the study used self-designed questionnaire what limited possibility to compare study results with previous research. Secondly, the study did not investigate participants’ psychological factors such as anxiety, depression, or coping strategies that may influence abilities to undertake training under stressful periods.

## Supplementary Information

**Additional file 1.** Impact of COVID-19 pandemic on Paralympic athletes.

## Data Availability

The datasets generated and/or analysed during the current study are available from the corresponding author on reasonable request.
