# Supplementary material for: Impact of COVID-19 pandemic on athletes with disabilities preparing for the Paralympic Games in Tokyo
Source: BMC Res Notes. 2021 Jun 14;14:233. doi: 10.1186/s13104-021-05646-0 (PMC8201434; doi:10.1186/s13104-021-05646-0)
Supplement: Supplementary file 1 — Additional file 1. Impact of COVID-19 pandemic on Paralympic athletes. [file 13104_2021_5646_MOESM1_ESM.docx]

**Additional file**

**Impact of COVID-19 pandemic on Paralympic athletes**

This research concerns assessment of impact of the COVID-19 pandemic on the functioning of Paralympians, in particular, their training capabilities. The results the study will be used by national and international Paralympic Committees to minimize the effects of the pandemic. Your personal data will not be kept anywhere. By completing the survey, you agree to participate in the research.

It will take 8-10 minutes to complete the survey.

1. Please state your sport’s discipline (check all that apply).

□ Archery

□ Athletics

□ Badminton

□ Boccia

□ Canoe

□ Cycling

□ Equestrian

□ Football 5-a-side

□ Goalball

□ Judo

□ Powerlifting

□ Rowing

□ Shooting Para sport

□ Sitting volleyball

□ Swimming

□ Table tennis

□ Taekwondo

□ Triathlon

□ Wheelchair basketball

□ Wheelchair fencing

□ Wheelchair rugby

□ Wheelchair tennis

□ Alpine skiing

□ Biathlon

□ Cross-country skiing

□ Para ice hockey

□ Snowboard

□ Wheelchair curling

□ Standing-beach-volleyball

1. Please state your sport’s class according to IPC Classification System.

_________________________________________________________

1. Please state your experience as Paralympic athlete (years).

_________________________________________________________

1. Have you undergone the COVID-19 test or were referred for it?

□ Yes

□ No

1. Have you been diagnosed with COVID-19 disease?

□ Yes

□ No

1. If "YES", please specify how many days in the last 4 weeks you have NOT trained (if applicable)?

_________________________________________________________

1. Have your coach been diagnosed with COVID-19 disease?

□ Yes

□ No

_________________________________________________________

1. If “YES”, how many days you did NOT have professional advices from your coach within last 4 weeks (if applicable)?

_________________________________________________________

1. According to your original training program, not affected by pandemic, how many hours per week you should train (please state)?

_________________________________________________________

1. During the last 4 weeks, on average, how many hours a week you trained (please state)?

_________________________________________________________

1. Did you have access to your typical training place e.g. sports hall, stadium within last 4 weeks (choose one answer)?

□ Yes, as usual

□ Yes, but limited

□ Yes, more than usual

□ Not at all

1. Have you been taking part in sport camp in the last 4 weeks (choose one answer)?

□ Yes

□ Not, it was not planed during that time

□ Not, it was canceled due to COVID-19

1. Where did you train during the last 4 weeks (check all that apply)?

□ Home

□ Outdoor

□ Sport facilities

1. Do you feel satisfied with amount of time spend for training in the last 4 weeks (choose one answer)?

□ Yes

□ Not, because of pandemic

□ Not, because of other reasons

1. Do you need assistance or assistive device in training, which you can't use now, because of pandemic?

□ Yes

□ Not

1. Have you been quarantined in the last 4 weeks?

□ Yes

□ No

**Personal data**

1. What year were you born (year)?

_________________________________________________________

1. Please indicate your gender.

□ Male □ Female

1. Where do you live?

□ Urban area □ Rural are

1. Please state time since your injury or illness (years).

_________________________________________________________

1. What is your current marital status (chose one answer)?

□ Single

□ Married

□ Cohabiting or in a partnership

□ Separated or divorced

□ Widowed

1. What is the highest level of education that you have completed (chose one answer)?

□ Primary

□ Lower secondary

□ Higher secondary

□ Post-secondary

□ Short tertiary

□ Bachelor or equivalent

□ Master or equivalent

□ Other

1. What is your current working situation (check all that apply)?

□ Working for wages or salary

□ Self-employed

□ Working as unpaid family member e.g., □ working in family business

□ Housewife / househusband

□ Student

□ Unemployed

□ Retired due to the health condition

□ Retired due to age

□ Other

1. Do you need assistance with your day-to-day activities at home or outside?

□ Yes

□ No

1. Please state your disability (check one answer):

□ Paraplegia

□ Tetraplegia

□ Spina bifida

□ Cerebral palsy

□ Amputation of one upper limb

□ Amputation of both upper limb

□ Amputation of one lower limb

□ Amputation of both lower limb

□ Multiple sclerosis

□ Muscular dystrophy

□ Heine-Medina disease (poliomyelitis)

□ Other (please state) ______________
